# Structure–function studies of a novel laccase-like multicopper oxidase from *Thermothelomyces thermophila* provide insights into its biological role

**DOI:** 10.1107/S2059798323004175

**Published:** 2023-06-16

**Authors:** Christos Kosinas, Anastasia Zerva, Evangelos Topakas, Maria Dimarogona

**Affiliations:** a Laboratory of Structural Biology and Biotechnology, Department of Chemical Engineering, University of Patras, Caratheodory 1, 26504 Patras, Greece; b Industrial Biotechnology and Biocatalysis Group, Biotechnology Laboratory, School of Chemical Engineering, National Technical University of Athens, 5 Iroon Polytechniou Street, 15772 Athens, Greece; c Laboratory of Enzyme Technology, Department of Biotechnology, School of Applied Biology and Biotechnology, Agricultural University of Athens, 75 Iera Odos Street, 11855 Athens, Greece; Station Biologique de Roscoff, France

**Keywords:** laccase-like multicopper oxidases, LMCOs, biocatalysts, crystal structure, *Thermothelomyces thermophila*, molecular docking

## Abstract

Multicopper oxidases are promiscuous biocatalysts with great potential for the production of industrial compounds. This study is focused on the elucidation of the structure–function determinants of a novel laccase-like multicopper oxidase from the thermophilic fungus *Thermothelomyces thermophila* (*Tt*LMCO1), which is capable of oxidizing both ascorbic acid and phenolic compounds and thus is functionally categorized between the ascorbate oxidases and fungal ascomycete laccases (asco-laccases). The crystal structure of *Tt*LMCO1, determined using an *AlphaFold*2 model due to a lack of experimentally determined structures of close homologues, revealed a three-domain laccase with two copper sites, lacking the C-terminal plug observed in other asco-laccases. Analysis of solvent tunnels highlighted the amino acids that are crucial for proton transfer into the trinuclear copper site. Docking simulations showed that the ability of *Tt*LMCO1 to oxidize *ortho*-substituted phenols stems from the movement of two polar amino acids at the hydrophilic side of the substrate-binding region, providing structural evidence for the promiscuity of this enzyme.

## Abstract 2

The crystal structure of a novel laccase-like multicopper oxidase from the thermophilic fungus *Thermothelomyces thermophila* was refined at 1.9 Å resolution. Ligand-docking simulations reveal conformational changes that influence substrate specificity.

## 1. Introduction

Laccase-like multicopper oxidases (LMCOs; EC 1.10.3.2; *p*-benzenediol:oxygen oxidoreductases) are categorized into the multicopper oxidase (MCO) superfamily along with ascorbate oxidases (EC 1.10.3.3), ferroxidases (EC 1.16.3.1), nitrite reductases (EC 1.7.2.1) and ceruloplasmins (EC 1.16.3.1). The members of the MCO superfamily can directly oxidize a broad range of phenolic compounds such as *ortho*- and *para*-diphenols, aminophenols, polyphenols, polyamines, aryl diamines and some inorganic ions (Solomon *et al.*, 1996 ▸; Gianfreda *et al.*, 1999 ▸; Schlosser & Höfer, 2002 ▸).

Like common laccases, LMCOs perform a four-electron oxidation of a wide variety of phenolic compounds with the concomitant reduction of a dioxygen molecule to two molecules of water. The mechanism of dioxygen reduction involves the extraction of four electrons from the oxidized substrates and the delivery of four protons to the active site. The reaction combines several steps into an intriguing and concerted mechanism which protects the enzyme from the generation of extensive free-radical species and inactivation (Hakulinen & Rouvinen, 2015 ▸). LMCO substrates of low molecular weight can be used as oxidative mediators for the indirect oxidation of larger compounds, polymers or recalcitrant compounds, expanding the total substrate range of MCOs (Bourbonnais & Paice, 1990 ▸; Morozova *et al.*, 2007 ▸), while rational or semi-rational engineering has been applied to further broaden the substrate range, a significant property for industrial biocatalysts (Mateljak *et al.*, 2019 ▸). Besides their established use in the food, paper and textile industries (Arregui *et al.*, 2019 ▸), recent studies have initiated a growing interest in novel biotechnological applications of LMCOs, such as in wastewater treatment, the synthesis of novel bioactive compounds and lignin degradation, as well as biosensor development (Moreno *et al.*, 2020 ▸; Ihssen *et al.*, 2014 ▸; Polak *et al.*, 2016 ▸; Zerva *et al.*, 2019 ▸; Zouraris *et al.*, 2020 ▸).

Although the MCO superfamily is significantly diverse, some characteristics are common to all members of the family. The overall fold is comprised of three cupredoxin-like domains, each of which has a Greek-key β-barrel topology (Giardina *et al.*, 2010 ▸). Two catalytic copper sites are identified: a type 1 (T1) copper site close to the region where substrate oxidation occurs, and a trinuclear cluster (TNC) formed by a single type 2 (T2) copper ion and a pair of type 3 (T3a and T3b) copper ions. An electron extracted from the oxidized molecule in the T1 site is shuttled through a sequence of three highly conserved amino acids (the HCH bridge) to the TNC, where reduction of a dioxygen molecule to water occurs (Arregui *et al.*, 2019 ▸). To date, most structure–function studies of laccases and LMCOs have focused on the residues forming these two copper sites. Glycosylation has also been thoroughly investigated and is considered to influence the stability and/or activity of fungal members of the family (Vite-Vallejo *et al.*, 2009 ▸; Maestre-Reyna *et al.*, 2015 ▸), which exhibit an extent of glycosylation ranging between 10% and 25% (mostly in basidiomycete laccases) or even greater than 30% in some ascomycete laccases (asco-laccases) (Shleev *et al.*, 2004 ▸; Ernst *et al.*, 2018 ▸).

Some MCOs are categorized into the AA1 family of the Carbohydrate Active EnZymes database (CAZy; https://www.cazy.org; Drula *et al.*, 2022 ▸), which is further divided into three subfamilies. Subfamily 1 comprises mainly laccases originating from basidiomycetes (considered ‘true’ laccases), subfamily 3 comprises laccases from ascomycetes and subfamily 2 includes ferroxidases and other LMCOs. To date, 77 biochemically characterized enzymes have been reported in the CAZy database, of which 25 have experimentally determined structures. Most of the known laccase structures originate from basidiomycetes and several structures are from plant (Xie *et al.*, 2020 ▸) and bacterial (Enguita *et al.*, 2003 ▸; Paavola *et al.*, 2021 ▸; Olmeda *et al.*, 2021 ▸) sources, while only five structures are been reported from ascomycetes.

In spite of significant research on structure–function relationships in LMCOs (Hakulinen *et al.*, 2002 ▸; Kallio *et al.*, 2011 ▸; Ernst *et al.*, 2018 ▸; Polyakov *et al.*, 2017 ▸, 2019 ▸), there are many questions that remain unanswered, such as the identity of the residues involved in dioxygen reduction and ligand binding, the determinants of the redox potential of the enzyme and even the effect of glycosylation on enzymatic stability and substrate specificity.

In this study, we report the crystal structure of an LMCO from the thermophilic fungus *Thermothelomyces thermophila* (*Tt*LMCO1), which was used to perform ligand-docking simulations and to correlate these results with biochemical findings. Although *Tt*LMCO1 is categorized into the Ascomycetes family, it exhibits substantial differences from other characterized LMCOs from Ascomycetes, while sharing some similarities with plant laccases and ascorbate oxidase from *Curcubita pepo*. *Tt*LMCO1 has a rather low redox potential (*E* ^0^), which is reflected in a relatively narrow substrate range compared with other bacterial or fungal laccases (Zerva *et al.*, 2019 ▸; Zouraris *et al.*, 2020 ▸). On the other hand, it is active against a broader substrate range compared with ascorbate oxidases, which only oxidize ascorbic acid and its derivatives, despite their high sequence similarity (Wimalasena & Dharmasena, 1994 ▸; Itoh *et al.*, 1995 ▸; Barberis *et al.*, 2014 ▸). The structural characteristics that differentiate *Tt*LMCO1 from other asco-laccases and also from plant MCOs are highlighted, providing evidence for its as yet unknown biological role.

## 2. Methods

### 2.1. Expression, purification and crystallization of *Tt*LMCO1

Recombinant *Tt*LMCO1 was expressed in *Pichia pastoris* X33 and deglycosylated as described previously (Zerva *et al.*, 2019 ▸). The copper-loaded enzyme was purified from the culture supernatant after supplementation of the culture with 0.025 m*M* copper(II) sulfate (Zerva *et al.*, 2019 ▸). Immobilized metal-affinity chromatography (IMAC) with Co^2+^ resin was applied to separate endoglycosidase H from the deglycosylated enzyme. A final polishing step was performed by size-exclusion chromatography on a 16/60 Sephacryl column. The purified enzyme was concentrated to 16 mg ml^−1^ in 20 m*M* Tris–HCl pH 8.0 and was submitted to crystallization trials using the JCSG-plus and PACT premier screening kits (Molecular Dimensions). Equal volumes (0.7 µl) of enzyme solution and reservoir solution were mixed in each well of a 96-well plate (SWISSCI) implementing the sitting-drop vapour-diffusion method. After 10–12 days, protein crystals with an irregular rod-like shape and a characteristic light blue colour appeared in several crystallization conditions, such as 0.2 *M* ammonium chloride, 0.1 *M* 2-(*N*-morpholino)ethanesulfonic acid (MES) pH 6.0, 20%(*w*/*v*) PEG 6000 (Supplementary Fig. S1*a* ). In an effort to obtain larger crystals, optimization was performed for this condition by adjusting the pH to 5.5 (Supplementary Fig. S1*b* ). For X-ray data collection, crystals were mounted on litholoops and transferred into mother liquor containing 20% glycerol as a cryoprotectant before being flash-cooled in liquid nitrogen. X-ray data were collected on beamline P13 at the PETRA III storage ring, DESY, Hamburg, Germany operated by EMBL Hamburg (Cianci *et al.*, 2017 ▸).

### 2.2. Structure determination

X-ray data were processed with *XDS* (Kabsch, 2010 ▸) and scaled with *AIMLESS* (Evans & Murshudov, 2013 ▸), which is included in the *CCP*4 suite (Agirre *et al.*, 2023 ▸). Although the *Tt*LMCO1 crystals diffracted to 1.3 Å resolution, data beyond 1.9 Å resolution were excluded to improve the data-processing statistics (the data-resolution cutoff criteria were 〈*I*/σ(*I*)〉 > 1.5 and CC_1/2_ > 0.5). The crystal was assigned to space group *P*4_3_, with unit-cell parameters *a* = *b* = 74.85, *c* = 118.99 Å and one molecule in the asymmetric unit. Data-collection statistics are shown in Table 1 ▸.

The closest homologue with an experimentally determined structure displayed low sequence identity to *Tt*LMCO1 (PDB entry 1aoz, with 28% sequence identity over 85% coverage; Messerschmidt *et al.*, 1992 ▸). Initial attempts to use it as a template for molecular replacement (MR) in *Phaser* (McCoy *et al.*, 2007 ▸) provided a solution with *R*_work_ and *R*_free_ values of 0.46 and 0.49, respectively (LLG = 56.09, TFZ = 6.8), while the resulting electron-density maps indicated several chain breaks and clashes with symmetry-related molecules. Further attempts to autobuild the protein model using *Buccaneer* (Cowtan, 2012 ▸) led to an improved model and decreased *R*_work_ and *R*_free_ to 0.38 and 0.39, respectively. However, subsequent rounds of manual model building and real-space and reciprocal-space refinement failed to further decrease the *R* factors. To address this, an *AlphaFold*2 (Jumper *et al.*, 2021 ▸) prediction was generated and was instead used as a model for MR. The predicted model was assessed according to the predicted aligned error plot and the confidence measure (pLDDT) for each residue (Supplementary Fig. S2). Regions of the predicted model with poor pLDDT score (pLDDT < 50) were omitted from the MR model; specifically, residues 1–15 and 179–251 as well as the C-terminal residues 601–630. Using this model for MR, *Phaser* (McCoy *et al.*, 2007 ▸) found a unique solution with high LLG (14139) and TFZ (107.5) scores. Manual model building and *N*-glycan addition were performed with *Coot* (Emsley *et al.*, 2010 ▸). Structure refinement was performed with *REFMAC* (Murshudov *et al.*, 2011 ▸). For cross-validation, 5% of the data were excluded from the refinement for *R*_free_ calculations (Brünger, 1992 ▸). Solvent molecules were added using *REFMAC* and inspected manually using *Coot*. H atoms were added at riding positions in the final step of the refinement. The quality of the final model was evaluated using *MolProbity* (Chen, Arendall *et al.*, 2010 ▸). Refinement statistics are shown in Table 2 ▸. The coordinates and structure factors have been deposited in the PDB with accession code 7zn6. Graphical display of the structure and analysis were performed with *PyMOL* 2.0 (Schrödinger).

### 2.3. Solvent-channel characterization

The *CAVER* 3.0 *PyMOL* plugin (Pavelka *et al.*, 2016 ▸) was used for solvent-channel identification. The starting position for each tunnel estimation was defined using the coordinates of the T2 Cu or the coordinates of the O1 atom of dioxygen as input for the T2 and T3 tunnels, respectively. Default values were used for tunnel estimation. The results for each tunnel were evaluated in terms of average tunnel bottleneck, average tunnel length and average tunnel throughput. The tunnels with the minimum tunnel length and the highest values for tunnel throughput were selected and presented.

### 2.4. Substrate screening

All substrates for investigation of the substrate scope of *Tt*LMCO1 were purchased from Merck KGaA (Darmstadt, Germany) or Thermo Fisher Scientific (Waltham, Massachusetts, USA) and were of the highest purity available. The ability of *Tt*LMCO1 to oxidize different substrates was explored in 24 h reactions. The tested substrates (2 m*M*) were mixed with the enzyme (0.1 U, measured using ABTS as a substrate, corresponding to 16 µg protein) in 50 m*M* phosphate–citrate buffer pH 5.0 and incubated for 24 h at 40°C and 800 rev min^−1^ in an Eppendorf thermomixer. The final volume of the reaction was 250 µl. The reaction parameters (*i.e.* substrate concentration and temperature) had already been optimized, as described in a previous publication (Zerva *et al.*, 2019 ▸). The UV–Vis spectra (250–750 nm) of the reactions and their respective controls with heat-inactivated enzyme were recorded after 15 min and 24 h of reaction using a polystyrene flat-bottom Greiner CELLSTAR 96-well plate (Greiner Bio-One GmbH, Austria) in a SpectraMax 250 microplate reader (Molecular Devices, California, USA) to determine differences in absorbance maxima. 1 U is defined as the amount of enzyme that oxidizes 1 µmol of substrate per minute.

### 2.5. Molecular-docking simulations

Docking simulations were performed in *YASARA Structure* (Krieger & Vriend, 2014 ▸). The ligand structures were retrieved from PubChem (Kim *et al.*, 2021 ▸). Ligand structures were cleaned and their hydrogen-bonding network was optimized (Krieger & Vriend, 2014 ▸). Their geometries were also optimized using semi-empirical quantum-mechanics calculations. The structure of *Tt*LMCO1 (PDB entry 7zn6) was used after removing all heteroatoms and waters. Polar H atoms were added and the hydrogen network was optimized before proceeding with docking simulations (Krieger *et al.*, 2012 ▸). All structures were curated with *AutoSMILES* (Jakalian *et al.*, 2002 ▸) before docking simulations. The charge assignment for ligands was calculated at pH 4.5, at which *Tt*LMCO1 displays the highest activity (Zerva *et al.*, 2019 ▸). The charge assignment for ferulic acid that was used in docking simulations for ascorbate oxidase from *C. pepo* was calculated at pH 6.0, at which the enzyme displays the highest activity (Itoh *et al.*, 1995 ▸). Global docking in a 10^3^ Å simulation cell defined around the binding site was performed employing *AutoDock Vina*, completing 25 runs per simulation (the default settings; Trott & Olson, 2010 ▸).

The results were clustered and evaluated based on binding energies, dissociation constants and ligand orientation. Ligand-docking poses away from the binding site or with inappropriate orientation were rejected. The results with the highest *YASARA* scores (binding energy) and the lowest *K*_d_ (in the micromolar range) were selected and discussed.

## 3. Results and discussion

### 3.1. Overall crystal structure of *Tt*LMCO1

**Figure 1:**
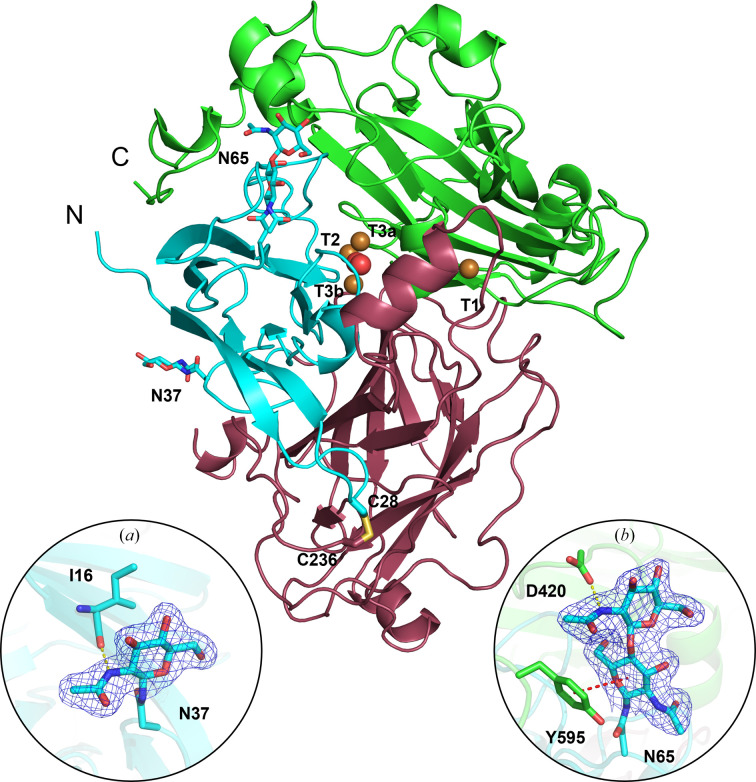
Cartoon representation of the *Tt*LMCO1 crystal structure. Each domain (A, B and C) is coloured differently: in cyan, raspberry and green, respectively. The disulfide bond between Cys28 and Cys236 is shown in stick representation. Copper ions and a dioxygen molecule located at the TNC are shown as brown and red spheres, respectively. Glycans are depicted as cyan sticks. (*a*) A NAG molecule is modelled into a 2*F*_o_ − *F*_c_ electron-density map contoured at 1σ. A hydrogen bond is formed by the main-chain carbonyl O atom of Ile16 and the N2 atom of NAG (yellow dotted line). (*b*) Two NAG molecules are modelled into a 2*F*_o_ − *F*_c_ electron-density map contoured at 1σ. A hydrogen bond is formed between the OD1 atom of Asp420 and the N2 atom of NAG (yellow dotted line). A CH–π interaction is formed between the phenyl group of Tyr595 and the d-glycopyranose ring of NAG (red dotted line).

The refined crystal structure of *Tt*LMCO1 (Fig. 1 ▸) contains 576 residues arranged in three cupredoxin-like domains. Domain A includes residues 11–134, domain B includes residues 135–372 and domain C includes residues 373–602. Residues 209–224 were not included in the final model due to insufficient electron density, similarly to the first ten N-terminal and the last 20 C-terminal residues. A disulfide bond is formed between Cys28 and Cys236, stabilizing the relative orientation of domains A and B.

A preliminary search using the *NetNGlyc* server proposed that *Tt*LMCO1 has three putative *N*-glycosylation sites (Asn37, Asn65 and Asn602; Gupta & Brunak, 2002 ▸). In accordance with the bioinformatic analysis, the crystal structure revealed two *N*-glycosylation sites, including Asn37 [one *N*-acetylglucosamine (NAG) molecule] and Asn65 (two NAG molecules). The N atom of the NAG residue on Asn37 forms a hydrogen bond to the backbone-carbonyl O atom of Ile16 (Fig. 1 ▸ *a*). In addition, the glycosylation on Asn65 seems to fix the relative positioning of domains A and C in the crystal lattice through the formation of a hydrogen bond between the N2 atom of the second NAG molecule and the OD2 atom of Asp420. Finally, a CH–π interaction between Tyr595 and the first NAG molecule leads to the stabilization of the C-terminus of the enzyme (Fig. 1 ▸ *b*).

The implications of *N*-glycosylation for the folding, stability and activity of laccases have been revealed in previous reports (Ernst *et al.*, 2018 ▸; Arregui *et al.*, 2019 ▸; Bento *et al.*, 2010 ▸). A recent crystallographic study of another asco-laccase from *T. thermophila* (*Mt*L) identified several glycosylation sites which are rather conserved in all known asco-laccase structures and suggested that *N*-glycans should contribute to the relative stabilization of protein domains A, B and C (Ernst *et al.*, 2018 ▸). Moreover, the study suggests that the solvent-exposed glycans could mediate the interaction of the laccase with large biopolymers or carbohydrate-rich substrates such as lignocellulose. *Tt*LMCO1 has considerably fewer *N*-glycosyl­ation sites compared with other asco-laccases; however, they still seem to contribute to conformational stability as discussed above. As shown in Supplementary Table S1, *N*-glycosylation on Asn37 is only conserved in *Mt*L (Asn61) and *Botrytis aclada* laccase (*Ba*L; Asn55), despite the fact that the asparagine residue is conserved in all asco-laccases, while Asn65 is not conserved in other asco-laccases.

A common feature of several MCOs is the development of a multimeric arrangement, as mentioned in several studies (Hakulinen & Rouvinen, 2015 ▸). Asco-laccases regularly form dimeric assemblies in a head-to-head arrangement of the T1 pockets of the enzyme (Ernst *et al.*, 2018 ▸). Moreover, a dodecameric arrangement of a bacterial MCO in its active form has recently been reported (Paavola *et al.*, 2021 ▸). Analysis of the *Tt*LMCO1 structure by the *Protein Interfaces, Surfaces and Assemblies* (*PISA*) server (Krissinel & Henrick, 2007 ▸) did not suggest dimer formation, while the interactions between *Tt*LMCO1 monomers are a result of crystal packing. The monomeric state of *Tt*LMCO1 was also corroborated by gel-filtration chromatography (data not shown).

To date, one MCO structure and four laccase structures from the Ascomycota family have been deposited in the PDB (Hakulinen & Rouvinen, 2015 ▸; Ernst *et al.*, 2018 ▸). These are laccases from *Thielavia arenaria* (*Ta*L; PDB entry 3pps), *Botrytis aclada* (*Ba*L; PDB entry 3sqr), *Melanocarpus albomyces* (*Ma*L; PDB entry 1gw0) and *Myceliopthora thermo­phila* (*Mt*L; PDB entry 6f5k) and an MCO from *Aspergillus niger* (*An*MCO; PDB entry 5lm8). Among these, the *DALI* server (Holm, 2020 ▸) only identified *Ta*L asco-laccase as a structural homologue, while all other hits displayed low sequence identity to *Tt*LMCO1 (Supplementary Fig. S3). The closest structural homologue, an ascorbate oxidase from *C. pepo* (PDB entry 1aoz), shares only 30% sequence identity with *Tt*LMCO1. Other structural homologues of *Tt*LMCO1 include five laccases from Basidiomycota species (*Metuloidea murashkinskyi*, *Trametes hirsuta*, *T. versicolor*, *Coprinus cinereus* and *Cerrena* sp.), a plant laccase from *Zea mays* and two bacterial laccases (from *Pediococcus acidilactici* and a marine bacterium of unknown classification) (Supplementary Table S2).

As mentioned in Section 2, the closest structural homologue (PDB entry 1aoz) failed to generate a robust MR model. Post analysis involving the superposition of the two experimentally determined structures revealed several differences, mainly in loop regions such as loop 382–426 in *C. pepo* ascorbate oxidase, which is much more elongated than the corresponding *Tt*LMCO1 loop, and further clashes in *Tt*LMCO1 loop 529–539. However, the most pronounced difference that could account for the unsuccessful MR attempts is located at the C-terminus of the two enzymes (Supplementary Fig. S4), a region of interest in *Tt*LMCO1 that is further analysed in Section 3.3. The *Tt*LMCO1 structure was thus solved using an *AlphaFold*2 prediction, which when superimposed with the final refined experimental *Tt*LMCO1 model exhibits an r.m.s.d. on C^α^ atoms of 1.3 Å, in contrast to *C. pepo* oxidase, which has an r.m.s.d. on C^α^ atoms of 2.5 Å. Superposition of the *AlphaFold*2 prediction with the final refined structure of *Tt*LMCO1 did not indicate any significant structural differences between the main-chain conformations of the two models, with the exception of the C-terminal region (Supplementary Fig. S4).

### 3.2. Copper sites

#### 3.2.1. T1 copper site

**Figure 2:**
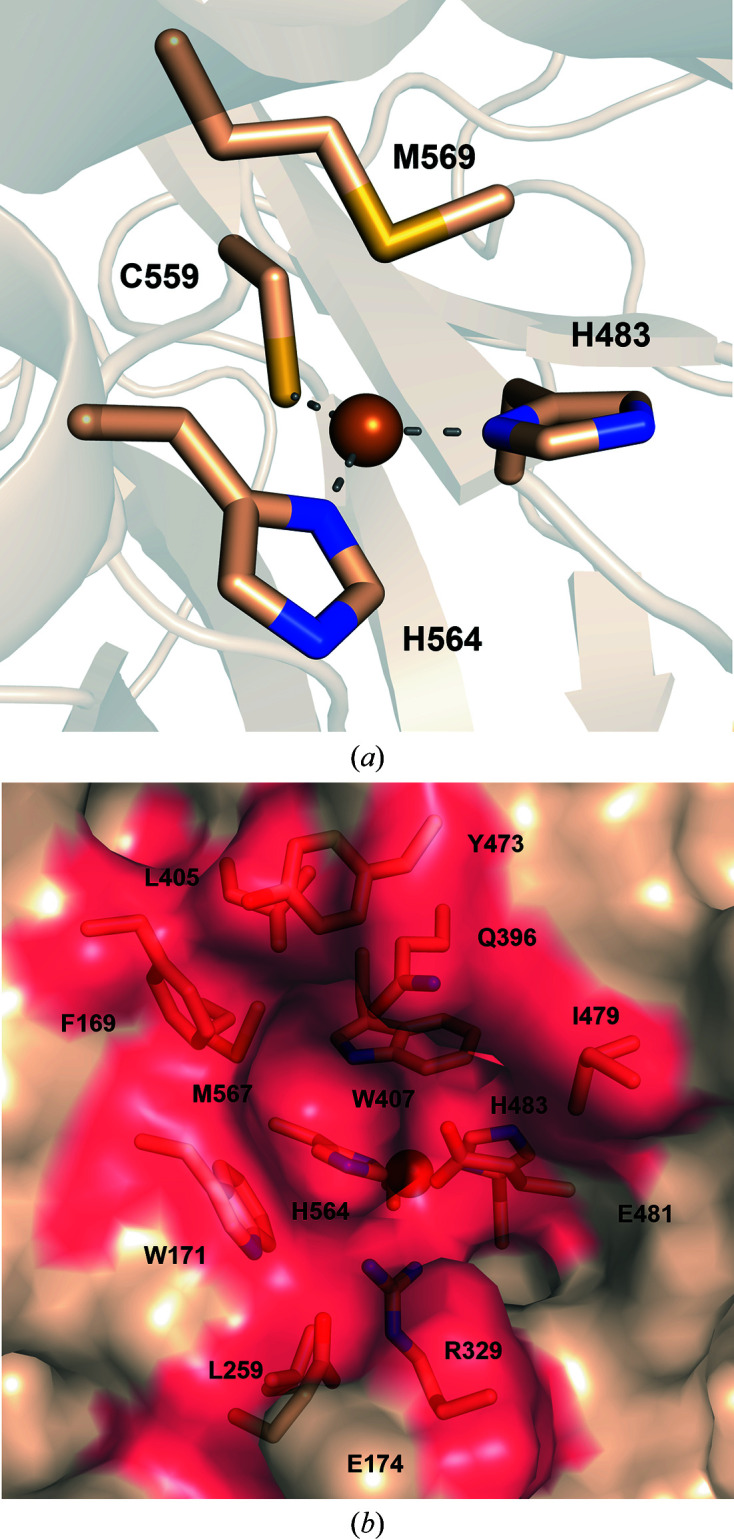
Representation of the substrate-binding site of *Tt*LMCO1. (*a*) T1 copper (sphere) with coordinating His483, Cys559 and His564 (sticks). Coordination bonds are shown as grey dashed lines. Met569 is axially positioned to the T1 copper. (*b*) Semi-transparent surface representation of the binding site in *Tt*LMCO1. The side chains of amino acids forming the substrate-binding site are shown as wheat sticks.

In the T1 site, the copper ion is coordinated by the ND1 atoms of His564 (2.1 Å distance) and His483 (2.0 Å distance) and by the thiolate group of Cys559 (2.2 Å distance), forming a trigonal plane. Also, a methionine residue (Met569) is positioned axially to the T1 copper at a distance of 3.15 Å (Fig. 2 ▸ *a*). The nature of the axially positioned residue has been correlated with the redox potential (*E* ^o^) of the enzyme in some studies (Rodgers *et al.*, 2010 ▸). The methionine residue identified in the *Tt*LMCO1 structure is typically found in laccases with low *E* ^o^ (<+460 mV) from plants and bacteria (Pardo & Camarero, 2015 ▸). However, reports of the redox potentials of other laccases do not support a conclusive correlation of redox potential and the nature of the axial T1 copper ligand (Ernst *et al.*, 2018 ▸). It is interesting to note that among the closest structural homologues, the only MCO with a methionine in the axial position is the ascorbate oxidase from *C. pepo*. The two enzymes share a low redox potential (Murata *et al.*, 2006 ▸), but they present significantly different biochemical properties and substrate specificity, supporting the idea that the biochemical properties of LMCOs and their substrate scopes are defined by multiple structural attributes.

According to known crystal structures of MCOs complexed with ligands, the substrate-binding pocket, where oxidation occurs, is adjacent to the T1 copper site (Hakulinen & Rouvinen, 2015 ▸). *Tt*LMCO1 was superposed onto the structure of 2,6-dimethoxyphenol (2,6-DMP)-bound *Ma*L (PDB entry 3fu7), which is the first reported structure of a ligand-bound asco-laccase (Kallio *et al.*, 2009 ▸). The *Tt*LMCO1 residues potentially forming the substrate-binding site include several hydrophobic residues (Phe169, Trp171, Leu405, Trp407, Tyr473 and Met567) and also charged amino acids (Glu481, Arg329 and Glu174 along with His564) (Fig. 2 ▸ *b*). According to the structure of 2,6-DMP-bound *Ma*L, His564 that is implicated in T1 copper coordination should contribute to substrate binding and is considered to be the primary electron acceptor of the oxidized substrate (Kallio *et al.*, 2009 ▸). In other asco-laccases, a carboxylate residue (Glu or Asp), perpendicular to the trigonal plane of the T1 copper, contributes to the polar recognition of the substrate (Hakulinen & Rouvinen, 2015 ▸; Supplementary Fig. S5). There is also one reported case of an asco-laccase in which the carboxylate residue is replaced by a histidine (Ernst *et al.*, 2018 ▸). In *Tt*LMCO1, a hydrophobic leucine residue (Leu259) is instead identified, similar to *C. pepo* and in contrast to other asco-laccases.

#### 3.2.2. Trinuclear copper site (TNC)

**Figure 3:**
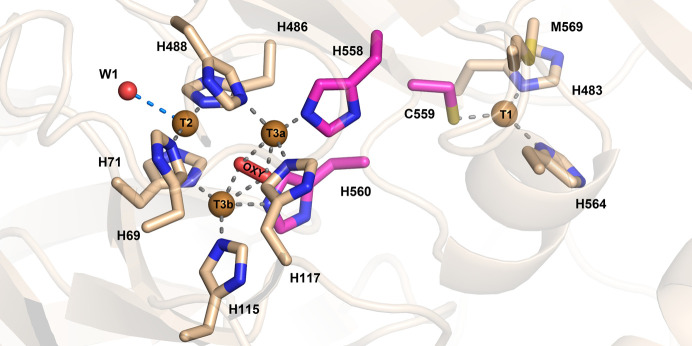
*Tt*LMCO1 copper sites. Copper ions in the TNC (left) are shown as brown spheres and are coordinated by histidine residues shown as wheat and magenta sticks. A dioxygen molecule is located between the T3 coppers, and a water molecule (W1) is modelled next to the T2 copper ion. Coordination bonds are shown as grey dashed lines, while the electrostatic interaction of W1 with T2 is shown as a marine dashed line. His558, Cys559 and His560, which form the ‘histidine bridge’ that enables electron transfer from the T1 copper site to the TNC, are shown as magenta sticks.

Similarly to other laccases, the trinuclear copper site (TNC) of *Tt*LMCO1 is formed by two T3 coppers along with one T2 copper coordinated by histidine residues. The electrons abstracted from the oxidized substrate at the T1 site are transferred to the TNC through a highly conserved His-Cys-His motif (His558-Cys559-His560 in *Tt*LMCO1) that is considered to act as an electron shuttle (Jones & Solomon, 2015 ▸; Fig. 3 ▸). A dioxygen molecule is modelled between the T3a and T3b coppers rather than a water molecule or a hydroxyl anion, since it fits more accurately into the ellipsoid shape of the electron density (Supplementary Fig. S6), while the peaks in the difference electron-density map disappear. The presence of a dioxygen molecule between the T3 coppers corresponds to the fully reduced state of the enzyme according to Polyakov *et al.* (2019 ▸). In addition to this oxygen molecule, the coordination sphere of the T3 coppers includes the N atoms of His117, His488 and His558 for the T3a copper and the N atoms of His71, His115 and His560 for the T3b copper. The T2 copper is linearly coordinated by the N atoms of His69 and His486 and forms charge–dipole interactions with W1 (Fig. 3 ▸). Interatomic distances between the T2 and T3 Cu atoms and the dioxygen molecule are shown in Supplementary Table S3. Extensive serial crystallography studies on basidio-laccase from *Steccherinum murashkinskyi* highlight that the reduction of copper ions can be induced by increasing the radiation dose on a protein crystal (Polyakov *et al.*, 2017 ▸, 2019 ▸). The linear coordination of the T2 copper by the N atoms of His69 and His486 is a strong indication that the T2 copper is present in the reduced state. Analysis of the metal-binding sites using the *CheckMyMetal* server verified the coordination geometry of the T2 copper ion (Gucwa *et al.*, 2023 ▸).

### 3.3. Solvent tunnels

Apart from electron transfer between the two copper sites, transportation of protons is also necessary to complete the reduction of the dioxygen molecule to two water molecules in the TNC. Solvent tunnels or channels have been reported both for basidiomycete laccases and asco-laccases, assisting proton transfer to the TNC of MCOs either by delivering protons to the T2 copper (T2 copper channels; Quintanar *et al.*, 2005 ▸) or by the direct dispatch of protons to the dioxygen molecule between the T3 coppers (T3 copper channels). The latter channels are commonly found in basidiomycete laccases (Wu *et al.*, 2018 ▸; Polyakov *et al.*, 2017 ▸). Residues with carboxylate side chains exposed on the surface of the solvent channels are considered to be involved in the mechanism of proton transfer (Enguita *et al.*, 2003 ▸; Chen, Durão *et al.*, 2010 ▸).

**Figure 4:**
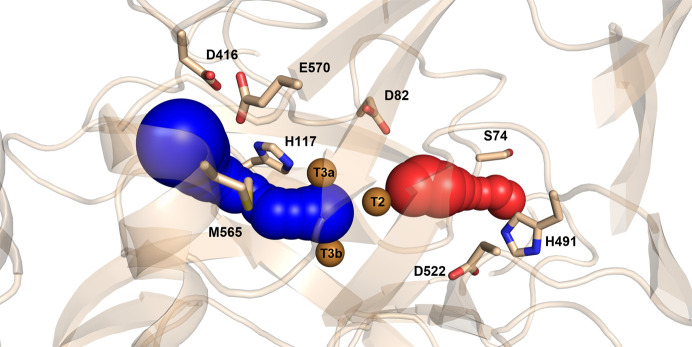
Graphical representation of *Tt*LMCO1 tunnels estimated by the *CAVER* 3.0 *PyMOL* plugin. The T2 and T3 tunnels are shown as red and blue spheres, respectively. Copper ions are shown as brown spheres and the side chains of the residues forming the tunnels are shown as wheat sticks.

The crystal structure of *Tt*LMCO1 was thus used to trace the solvent tunnels providing access to the trinuclear site. The predominant results from *CAVER* 3.0 analysis of the pathway of the T2 and T3 tunnels are shown in Fig. 4 ▸. The average length of the T2 tunnel is estimated at 8.2 Å, while the lowest bottleneck radius is calculated at 1.03 Å (Table 3 ▸). Asp82 and Asp552 form the inner part of the T2 tunnel, while His491 and Ser74 are located at the entrance to the tunnel. The average length of the T3 tunnel is estimated at 8.1 Å, while the lowest bottleneck radius is calculated as 0.98 Å (Table 3 ▸). The side chains of His117, Met565, Glu570 and Asp416 are exposed to the solvent inside the T3 tunnel.

**Figure 5:**
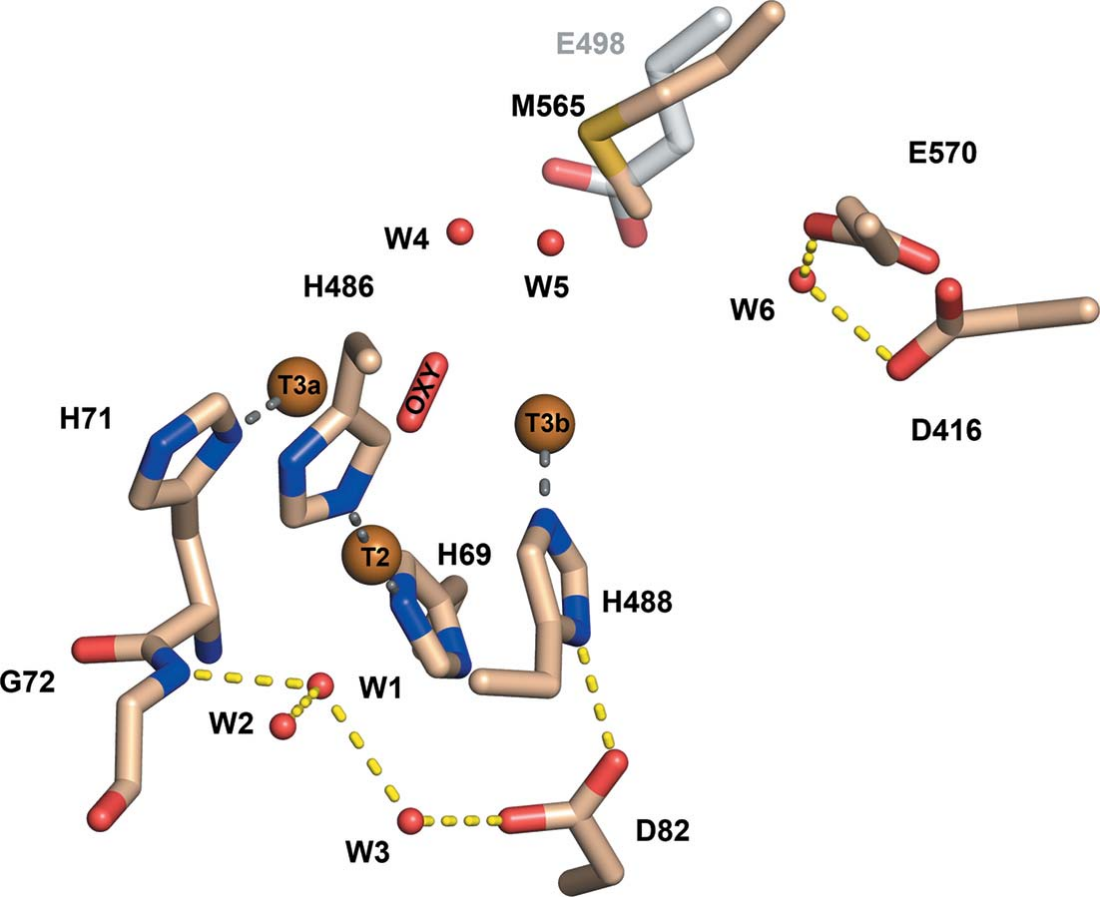
Detailed view of the TNC. Water molecules are shown as red spheres, hydrogen bonds as yellow dashed lines and coordination bonds as grey dashed lines. A water network (W4, W5 and W6) in the T3 channel is formed with the assistance of the hydrophilic side chain of Glu570 and Asp416. A methionine residue (Met565) is shown in place of Glu498 that is present in the CotA laccase from *Bacillus subtilis* (grey sticks).

The T2 tunnel has previously been suggested to participate in proton transfer to the TNC (Quintanar *et al.*, 2005 ▸), as well as in copper ion restoration in T2-depleted enzyme (Osipov *et al.*, 2015 ▸). In *Tt*LMCO1, the entrance to the T2 tunnel is partially blocked by His491, which potentially acts as a gate residue (Fig. 4 ▸). The low bottleneck radius of the T2 tunnel compared with the radius of a water molecule (1.03 Å) indicates that conformational changes are necessary for molecule transfer. Furthermore, inside the T2 tunnel a water molecule (W1) forms hydrogen bonds to the N atom of the backbone of Gly72 and water molecules W2 and W3 (Fig. 5 ▸). The side chain of Asp82 contributes to the stabilization of this network by interacting with W3 and the ND1 atom of His488 (Fig. 5 ▸). Polyakov and coworkers suggest that this aspartic acid residue is present in many laccase structures, contributing to the coordination of the water molecule that interacts with the T2 copper. W1 is 2.77 Å away from the T2 copper, which is too far for a coordination bond to occur. In the oxidized state of the enzyme, however, this water is assumed to form a coordination bond with the T2 copper, enabling planar four-coordination of the copper, which leads to subsequent cleavage of the O—O bond (Polyakov *et al.*, 2017 ▸).

Oxygen and water molecules are transferred through the T3 tunnel to the TNC. Several studies, mostly on basidiomycete laccases, support the presence of a carboxylate residue in the T3 channel that aids the transfer of protons to the dioxygen molecule between the T3 coppers. Mutational studies on the CotA laccase from *Bacillus subtilis* indicate a major impact of Glu498 on the catalytic mechanism of the enzyme, implying involvement of this residue in the direct transfer of a proton at the dioxygen molecule (Chen, Durão *et al.*, 2010 ▸). A glutamate residue is generally conserved in this position in other basidio-laccases (Bento *et al.*, 2010 ▸). In *Tt*LMCO1, a methionine (Met565) is present at this position instead of glutamate (Fig. 5 ▸). However, another glutamate (Glu570) is located at the entrance to the T3 tunnel next to an aspartic acid residue (Asp416). A water molecule (W6) is coordinated by the O atoms of the two side chains and participates in a network of water molecules penetrating the T3 channel (W4, W5 and W6). This supports the statement of Polyakov and coworkers that the nature of the negatively charged hydrophilic groups (Glu or Asp) assists the formation of the T3 water channel through the coordination of water molecules that penetrate deeply into the TNC (Polyakov *et al.*, 2017 ▸, 2019 ▸).

### 3.4. Role of the C-terminus in *Tt*LMCO1

A common feature of reported asco-laccase structures is that the C-terminus blocks the T3 tunnel that gives access to the TNC of the enzyme (Kallio *et al.*, 2009 ▸; Ernst *et al.*, 2018 ▸; Hakulinen *et al.*, 2002 ▸; Ferraroni *et al.*, 2017 ▸; Osipov *et al.*, 2014 ▸). Indeed, superposition of the *Ba*L*, Ta*L, *Ma*L, *Mt*L and *An*L structures reveals a conserved four-amino-acid sequence (DSGL/I) that penetrates the T3 tunnel and is referred to as the C-plug. Multiple sequence alignment of *Tt*LMCO1 with asco-laccases reveals a similar motif at its C-terminus (DSGH; Supplementary Fig. S7). The most important difference compared with other asco-laccases is that the final amino acid is a hydrophilic histidine and not an aliphatic leucine or isoleucine. Mutation of the terminal leucine to an alanine in *Ma*L resulted in breakage of the hydrogen bond between the C-terminus of the enzyme and the T3 copper-coordinating His140 (Andberg *et al.*, 2009 ▸). Despite the slightly altered conformation of the TNC, the activity of the enzyme towards 2,6-DMP did not change significantly and the redox potential of the enzyme also remained unaffected. Given that the terminal leucine is conserved among asco-laccases, *Tt*LMCO1 could represent a naturally occurring mutant with a modified C-terminus, resulting in a modified, but still functional, TNC.

**Figure 6:**
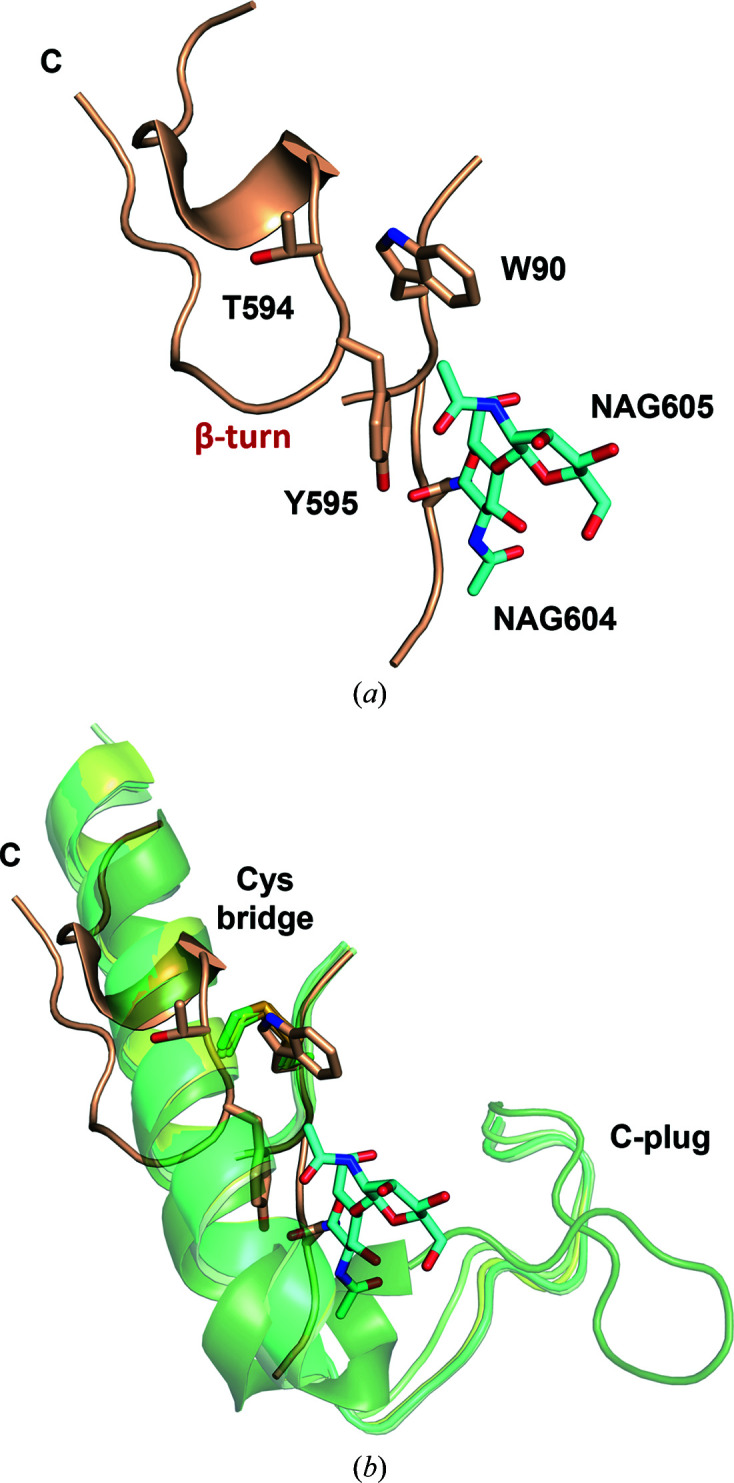
(*a*) Cartoon representation of the C-terminus of *Tt*LMCO1. The side chains of Trp90, Thr594 and Tyr595 are shown as wheat sticks. Two NAG molecules linked to Asn65 are shown as cyan sticks. (*b*) Superposition of the C-terminus of *Tt*LMCO1 with the C-termini of other asco-laccases (*Ba*L, *Ta*L, *Ma*L, *Mt*L and *An*L) shown in cartoon representation. *Tt*LMCO1 is coloured wheat, while the other asco-laccases are coloured shades of green. A cysteine bridge shown as sticks fixes the position of an α-helix at the C-terminus, allowing the formation of the C-plug.

Furthermore, since the last 20 amino acids of *Tt*LMCO1 were not included in the final structure due to a lack of electron density, there is no indication that the C-terminus of *Tt*LMCO1 penetrates the T3 tunnel. However, superposition of the *Tt*LMCO1 structure with those of the aforementioned asco-laccases highlights some interesting structural differences. A disulfide bridge stabilizes the position of an α-helix of the third domain with respect to the first domain of asco-laccases (Fig. 6 ▸ *b*). In *Tt*LMCO1 the cysteine residues are replaced by a tryptophan (Trp90, domain A) and a threonine (Thr594, domain C) (Fig. 6 ▸). Moreover, the C-terminal loop makes a β-turn after Tyr595, possibly forced by the two NAG molecules from glycosylation on Asn65 that interact with Tyr595 and that are absent in other asco-laccases. This may affect the ability of the C-terminus to act as a plug for the T3 channel, as opposed to the other asco-laccases, in which the C-terminus is oriented towards the T3 tunnel (Fig. 6 ▸ *b*).

### 3.5. Activity of *Tt*LMCO1 against a variety of laccase substrates

Although the substrate specificity of *Tt*LMCO1 for various common laccase substrates was determined in a previous study (Zerva *et al.*, 2019 ▸), a more thorough exploration of the substrate scope of the enzyme was performed in this work. Due to a lack of available data on the molar absorptivities of the oxidized species for many LMCO substrates, their oxidation was only assessed based on absorbance differences before and after incubation with *Tt*LMCO1, and the results are shown in Table 4 ▸. It has to be noted that since the oxidized products of each tested compound might have significantly variable absorbance properties, the results should be treated as qualitative. The substrate scope of *Tt*LMCO1 verifies our previous results and confirms that the promiscuity of the enzyme clearly distinguishes it from ascorbate oxidases, since these enzymes are not able to oxidize phenolic compounds. It is also shown that the existence of an *ortho*-hydroxy group is crucial for oxidation: pyrogallol and catechol, which contain *ortho*-hydroxy groups, are readily oxidized, but this is not the case for resorcinol and hydroquinone, which contain *meta*- and *para*-hydroxy groups, respectively. The same is also true for the existence of a methoxy group in an *ortho*-position, since 2,6-DMP, guaiacol and ferulic and caffeic acids are also rather easily oxidized compared with the corresponding unsubstituted compounds. This is in accordance with the rather low *E* ^0^ of the enzyme: the presence of electron-donating groups, such as hydroxy and methoxy groups, on the phenol ring decreases the *E* ^0^ of the compound [for example, the *E* ^0^ of catechol is 0.53 V versus the normal hydrogen electrode (NHE), compared with an *E* ^0^ of >0.8 V versus the NHE for phenol] and thus makes it more readily oxidized (Subrahmanyam *et al.*, 1991 ▸). Moreover, aromatic amines are easily oxidized by *Tt*LMCO1, similarly to most laccases. In the case of l-DOPA oxidation, a precipitate was formed after 24 h reaction, indicating polymerization of the substrate. This is common for most ascomycete laccases, since they are naturally implicated in melanin biosynthesis and in pigment formation in general (Janusz *et al.*, 2020 ▸; Sapmak *et al.*, 2015 ▸).

### 3.6. Molecular docking reveals residues that are involved in substrate recognition

Structural data on laccases or MCOs complexed with ligands are scarce; therefore, little is known about the residues involved in substrate recognition and conformational changes of the binding site. As shown above, *Tt*LMCO1 not only oxidizes ascorbic acid and its derivatives, but also phenolic substrates, with a higher activity towards those that bear methoxy or hydroxyl substituents at the *ortho* position of the phenol ring (Zerva *et al.*, 2019 ▸). Therefore, in addition to ascorbic acid, two further *Tt*LMCO1 substrates were selected for docking simulations: one with a single methoxy substitution at the *ortho* position (ferulic acid) and one with methoxy substitutions at both *ortho* positions (2,6-DMP). Global docking of the selected ligands around the residues that form the substrate site was performed using *YASARA* (Land & Humble, 2018 ▸). Docking results, in terms of calculated binding energy (kcal mol^−1^), dissociation constant (*K*_d_) and contact receptor residues, are presented in Supplementary Table S4.

Simulations for all ligands were initially performed with all protein atoms fixed. This strategy provided acceptable docking results for l-ascorbic acid, while docking simulations for the phenolic compounds resulted in high dissociation constants (over 200 µ*M*) and low binding energies. Therefore, several docking simulations were repeated for 2,6-DMP and ferulic acid enabling free movement of residue side chains that form the substrate-binding site. Eventually, after allowing free movement of only the side chains of Arg329 and Glu481, 2,6-DMP and ferulic acid were docked at catalytically relevant positions with acceptable scores. A more detailed outline of the docking poses and clustered results is presented in Appendix *A* [App appa].

**Figure 7:**
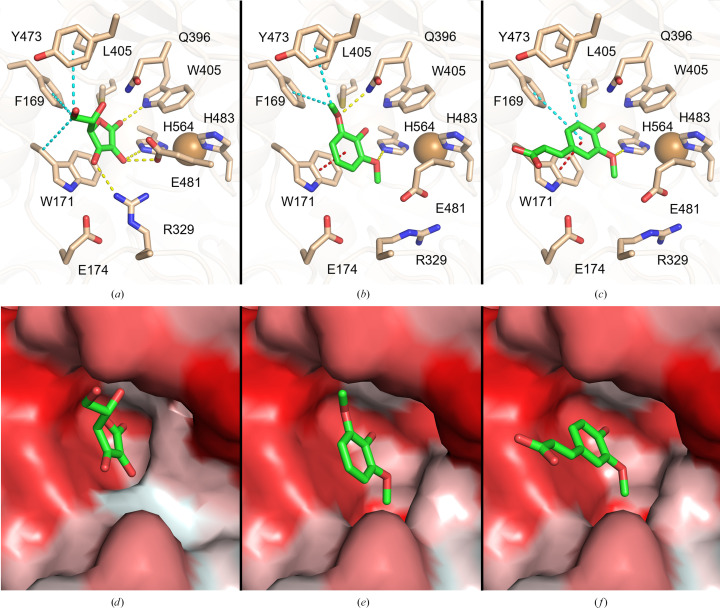
Graphical representation of docking-simulation results for *Tt*LMCO1 with l-ascorbic acid (*a*, *d*), 2,6-DMP (*b*, *e*) and ferulic acid (*c*, *f*). (*a*, *b*, *c*) Stick representations of the substrate-binding pocket of *Tt*LMCO1 with l-ascorbic acid, 2,6-DMP and ferulic acid, respectively. Hydrogen bonds are shown as yellow, hydrophobic interactions as cyan and π–π interactions as red dashed lines. (*d*, *e*, *f*) Surface representation of the substrate-binding pocket of *Tt*LMCO1 with l-ascorbic acid, 2,6-DMP and ferulic acid, respectively. Surface colouring of *Tt*LMCO1 varies from hydrophobic regions (red) to hydrophilic regions (white).

Ligand-binding positions in all examined cases indicate the placement of the O2 atom of ascorbic acid and 2,6-DMP, as well as the O3 atom of ferulic acid, within electron-transfer distance of the NE2 atom of His564 (Figs. 7 ▸ *a*, 7 ▸ *b* and 7 ▸ *c*), which is considered to be the direct acceptor of electrons from the oxidized substrate (Kallio *et al.*, 2009 ▸; Mehra *et al.*, 2018 ▸). This agrees with other known structures of three-domain laccases complexed with ligands [*Ma*L (PDB entry 3fu7), *Trametes trogii* laccase (PDB entry 2hrg) and *T. versicolor* laccase (PDB entry 1kya)]. A hydrophobic cavity of the binding pocket composed of Phe169, Trp171, Leu405, Trp407, Tyr473 and Met567 contributes to substrate binding via hydrophobic or π–π interactions in all three cases (Figs. 7 ▸ *a*, 7 ▸ *b* and 7 ▸ *c*). Docking of l-ascorbic acid into the substrate-binding site is further mediated by three hydrogen bonds to Arg329, Trp405 and Glu481, in addition to His564 which is common to all substrates (Fig. 7 ▸ *a*). Binding of 2,6-DMP is assisted by the formation of two hydrogen bonds, one to Gln396 and one to His564 (Fig. 7 ▸ *b*), while ferulic acid only forms a hydrogen bond to His564 (Fig. 7 ▸ *c*).

The interaction of methoxy groups of phenolic compounds with the NE2 atom of His564 is important for the electron-transfer pathway in laccases and agrees with other computational studies on laccases (Mehra *et al.*, 2018 ▸). Mehra and coworkers showed that in the absence of an *ortho*-positioned hydroxyl or methoxy group, the hydrogen bond to His458 (His564 in *Tt*LMCO1) is disrupted and therefore the phenolic hydroxy group of the substrate is located too far away to interact with His458. This is also in accordance with the biochemical data for *Tt*LMCO1 and also for most laccases, which are unable to oxidize substrates with a phenolic structure (Table 4 ▸) but readily oxidize compounds with a catecholic structure.

Moreover, the dissociation constant and binding energy for ferulic acid (Supplementary Table S4) indicate a higher affinity for this ligand compared with 2,6-DMP. This preference could be attributed to the architecture of the binding site: the single methoxy group of ferulic acid is more easily accommodated in the substrate cavity compared with the bulkier 2,6-DMP, which contains two opposing methoxy groups. Also, one side of the cavity is rather hydrophilic and is able to interact with hydroxy-bearing compounds, but the other side is lined with hydrophobic residues which, as shown from our docking results, are more likely to interact with the phenolic ring of the substrate through π–π stacking or hydrophobic interactions.

Conformational changes of the residues forming the binding site seem to be necessary to achieve phenolic ligand docking with favourable scoring. The side chains of Arg329 and Glu481 shift from their initial conformation, allowing enlargement of the binding pocket to fit catecholic or even pyrogallolic substrates with substitutions at the *ortho* positions (Figs. 7 ▸ *b* and 7 ▸ *c*). Indeed, initial attempts to dock 2,6-DMP and ferulic acid into the substrate-binding site while setting all residues at fixed positions did not result in an energetically and catalytically favourable solution. Since *Tt*LMCO1 readily oxidizes *ortho*-substituted phenolic compounds, it might be safe to assume that this conformation could be close to the actual positioning of these residues during catalysis.

The conformational flexibility of Arg329 and Glu481 could be related to the ability of *Tt*LMCO1 to act as a laccase rather than as an ascorbate oxidase. *Tt*LMCO1 shares a similar substrate-binding site architecture with ascorbate oxidase from *C. pepo* (Supplementary Fig. S8) as well as sharing a similar redox potential with plant oxidases; however, as a fungal LMCO it is able to oxidize a wide spectrum of phenolic compounds. Attempts to perform docking of ferulic acid to the structure of ascorbate oxidase from *C. pepo* implementing the same strategy as used for *Tt*LMCO1 did not provide favourable results (Supplementary Table S5). It could be possible that steric hindrance does not favour a shift of the side-chain conformations of Arg285 and Glu443 in ascorbate oxidase and thus hampers ligand binding to the substrate site.

*Tt*LMCO1 is the second laccase-like enzyme to be reported from *T. thermophila* and shows significantly different properties to the first laccase described from this organism (Berka *et al.*, 1997 ▸). Although many LMCOs have been found in fungal genomes and secretomes, this group of enzymes has not been studied in detail and thus the biological role of such enzymes in their natural hosts remains largely unexplained. Many enzymes with different kinetic and electrochemical properties, and the ability to oxidize different compounds, may participate in biological processes such as pigment synthesis and the decomposition of xenobiotics or even lignin in natural habitats. *Tt*LMCO1 closely resembles *C. pepo* ascorbate oxidase, but its biochemical properties mostly correlate with those of laccases. Thus, the present analysis leads to the hypothesis that *Tt*LMCO1 might correspond to an intermediate between plant ascorbate oxidases and microbial laccases, which are largely different from plant laccases. One such enzyme is an MCO from *Aspergillus terreus*, named TerA (Zaehle *et al.*, 2014 ▸), that shares high sequence similarity with *Tt*LMCO1. TerA belongs to the metabolic gene cluster of this organism for the biosynthesis of terrein, a molecule with diverse biological activities, but the exact role of this MCO in terrein biosynthesis has not been experimentally elucidated. Combined with the potential of *Tt*LMCO1 to oxidize natural compounds, such as epinephrine, l-DOPA and others, this could be an indication that these enzymes are involved in the production of pigments and other bioactive compounds in their natural hosts.

## 4. Conclusions

In this study, we report the crystal structure of an LMCO from the thermophilic fungus *T. thermophila*, which is an enzyme with no close structural homologues. As a three-domain laccase, the structure of *Tt*LMCO1 indicates that the enzyme combines distinct characteristics of different members of the MCO superfamily. *Tt*LMCO1 shares a similar substrate-binding site architecture with ascorbate oxidase from *C. pepo*. At the same time, as a fungal LMCO, *Tt*LMCO1 is able to oxidize a wide spectrum of phenolic compounds. Docking simulations with substrates that are oxidized by *Tt*LMCO1 provide evidence that the substrate specificity of these metalloproteins is not exclusively related to their redox potential but also to the architecture of the binding site and the side-chain flexibility of specific amino acids.

## 5. Related literature

The following reference is cited in the supporting information for this article: Robert & Gouet (2014 ▸).

## Supplementary Material

PDB reference: laccase-like multicopper oxidase, 7zn6

Supplementary Tables and Figures. DOI: 10.1107/S2059798323004175/jc5058sup1.pdf

## Figures and Tables

**Figure 8:**
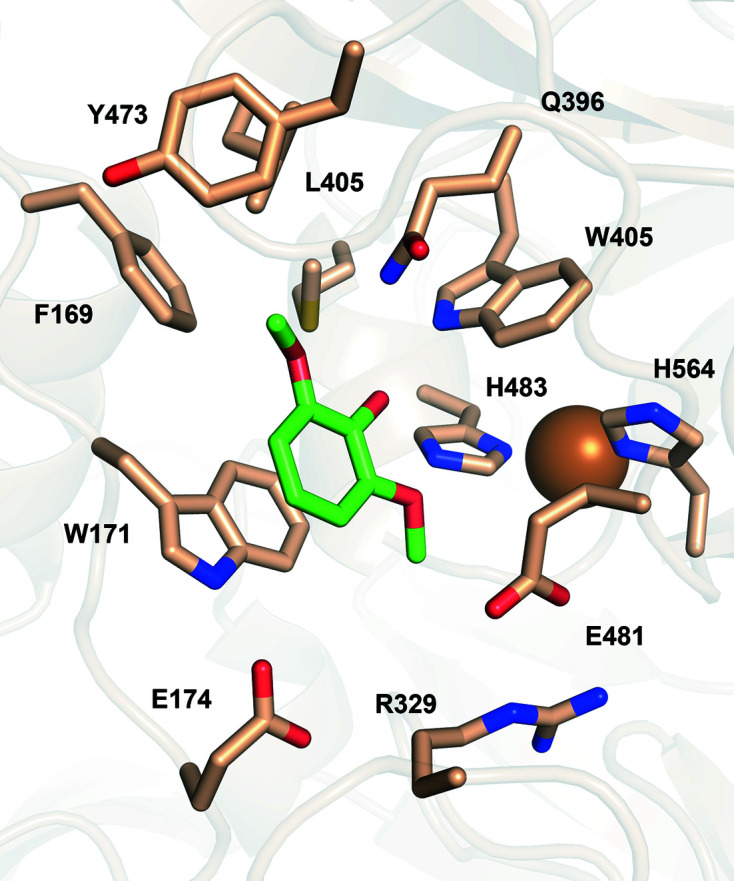
The three binding poses resulting from docking ascorbic acid into the *Tt*LMCO1 structure. A representative of the first cluster is shown as green sticks, while representatives of the second and third clusters are shown as yellow and cyan sticks, respectively.

**Figure 9:**
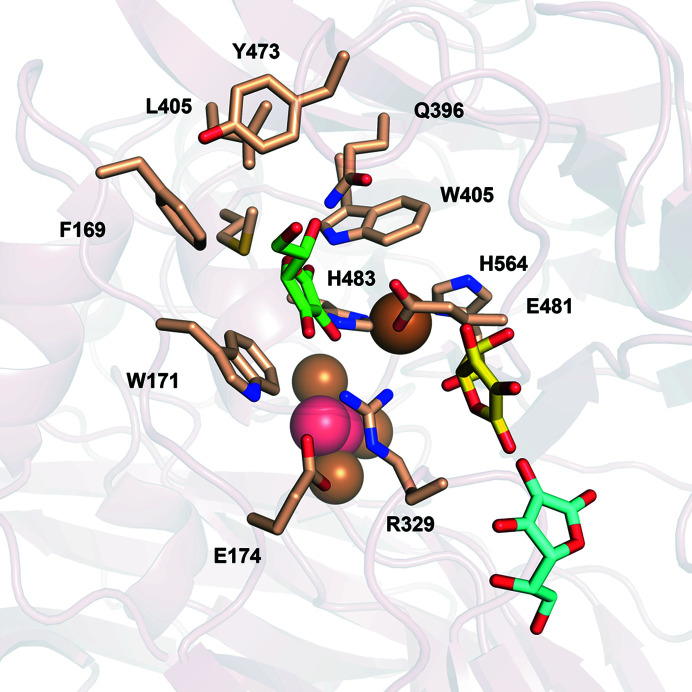
2,6-DMP docked into *Tt*LMCO1. The ligand molecule is presented as green sticks.

**Figure 10:**
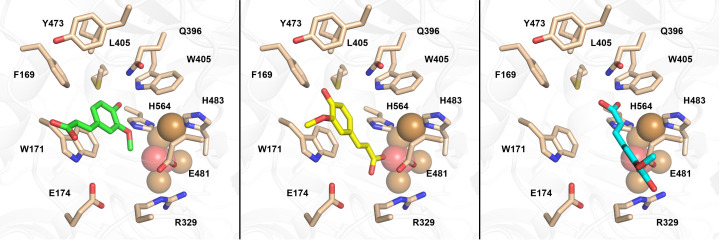
Binding poses of ferulic acid for *Tt*LMCO1. Left: ferulic acid corresponding to the first cluster is shown as green sticks. Middle: ferulic acid corresponding to the second cluster is shown as yellow sticks. Right: ferulic acid corresponding to the third cluster is shown as cyan sticks.

**Table 1:** Data-collection and processing statistics Values in parentheses are for the outer shell.

Diffraction source	P13, PETRA III
Wavelength (Å)	0.9763
Temperature (K)	100
Detector	EIGER R 4M
Crystal-to-detector distance (mm)	252.961
Rotation range per image (°)	0.05
Total rotation range (°)	360
Exposure time per image (s)	0.05
Space group	*P*4_3_
*a*, *b*, *c* (Å)	74.86, 74.86, 118.95
α, β, γ (°)	90, 90, 90
Mosaicity (°)	0.24
Resolution range (Å)	74.97–1.90
Total No. of reflections	664665 (39344)
No. of unique reflections	51534 (3299)
Completeness (%)	100 (100)
Half-set correlation CC_1/2_	0.999 (0.866)
Multiplicity	12.9 (11.9)
〈*I*/σ(*I*)〉	14.8 (2.9)
*R*_merge_	0.105 (1.028)
*R*_r.i.m._	0.114 (1.126)
*R*_p.i.m._	0.043 (0.456)
Overall *B* factor from Wilson plot (Å^2^)	30.59

**Table 2:** Structure solution and refinement Values in parentheses are for the outer shell.

Resolution range (Å)	74.97–1.90 (1.949–1.900)
Completeness (%)	100 (100)
σ Cutoff	none
No. of reflections, working set	48919 (3625)
No. of reflections, test set	2567 (181)
Final *R*_cryst_	0.152 (0.24)
Final *R*_free_	0.185 (0.26)
No. of non-H atoms	
Protein	4552
Ion	4
Ligand	76
Water	345
Total	4977
R.m.s. deviations	
Bond lengths (Å)	0.012
Angles (°)	1.700
Average *B* factors (Å^2^)	
Protein	37.23
Ion	29.48
Ligand	54.83
Water	42.25
Ramachandran plot	
Most favoured (%)	96.50
Allowed (%)	3.32

**Table 3:** Tunnel characteristics as estimated by the *CAVER* 3.0 *PyMOL* plugin Bottleneck radius is an estimation of the narrowest radius identified in the tunnel, while throughput reflects the predicted ability of a tunnel to transport small molecules.

Tunnel	Bottleneck radius (Å)	Length (Å)	Curvature (Å)	Throughput
T2	1.03	8.24	1.11	0.62
T3	0.98	8.16	1.18	0.76

**Table 4:** Substrate-oxidation spectrum of *Tt*LMCO1 Activity, in terms of the absorbance difference in the recorded UV–Vis spectrum between reaction and blank (recorded after reaction times of 15 min and 24 h), is indicated. The absorbance difference was determined at the wavelength where the maximum absorbance of the oxidized product was observed, as indicated in parentheses. –, substrates with no observed absorbance differences in the whole spectrum.

	Substrate (wavelength)	Δ*A*_15 min_	Δ*A*_24 h_
Hydroxybenzenes	Phenol	—	—
	Catechol (400 nm)	0.278	1.5
	Resorcinol	—	—
	Hydroquinone (400 nm)	—	0.084
	Pyrogallol (420 nm)	0.981	2.17
Methoxyphenols	Guaiacol (480 nm)	0.067	0.27
	2.6-Dimethoxyphenol (468 nm)	0.689	0.105 (precipitation)
Aromatic alcohols	3.4-Dimethoxybenzyl alcohol (400 nm)	—	0.032
Phenethyl alcohols	Tyrosol	—	—
Aromatic amines	*N*,*N*,*N*′,*N*′-Tetramethylphenylenediamine (610 nm)	2.6	3.5
	Epinephrine (482 nm)	0.124	0.78
	L-DOPA (476 nm)	0.063	Precipitation
Phenolic aldehydes	Vanillin	—	
Flavonoids	Catechin (442 nm)	0.623	2.37
	(+)-Epicatechin (390 nm)	0.846	3.3
Hydroxycinnamic acids	Caffeic acid (390 nm)	0.157	1.437
	Ferulic acid (380 nm)	0.083	0.576
	*p*-Coumaric acid	—	—
Hydroxybenzoic acids	Vanillic acid	—	—
	Gallic acid (382 nm)	0.948	2.36
	Protocatechuic acid (354 nm)	0.212	1.548
Aromatic azo compounds	ABTS (420 nm)	3.5	3.5
Other acids	Cinnamic acid	—	—

**Table 5:** *YASARA* clustered results for L-ascorbic acid docking simulations, giving the binding energy, the dissociation constant and the residues of *Tt*LMCO1 that contribute to ligand binding

Cluster No.	Binding energy (kcal mol^−1^)	Dissociation constant (µ*M*)	Contact receptor residues
1	6.37	21.6	Phe169, Trp171, Glu174, Arg329, Gln396, Leu405, Trp407, Tyr473, Gly475, Ala476, Glu481, His564, Met567
2	4.42	576.5	Asp328, Arg329, Pro330, Ile479, Val480, Glu481, Thr482, Arg528
3	4.29	743.9	Thr326, Arg327, Asp328, Arg329, Pro330, Pro332, Arg528

**Table 6:** *YASARA* clustered results for 2,6-DMP docking simulations, depicting the binding energy, the dissociation constant and the residues of *Tt*LMCO1 that contribute to ligand binding

Cluster No.	Binding energy (kcal mol^−1^)	Dissociation constant (µ*M*)	Contact receptor residues
1	5.46	99.5	Phe169, Trp171, Glu174, Leu259, Arg329, Pro330, Gln396, Leu405, Trp407, Tyr473, Ala476, Glu481, Ile561, His564, Met567

**Table 7:** *YASARA* clustered results for ferulic acid docking simulations, depicting the binding energy, the dissociation constant and the residues of *Tt*LMCO1 that contribute to ligand binding

Cluster No.	Binding energy (kcal mol^−1^)	Dissociation constant (µ*M*)	Contact receptor residues
1	6.39	20.7	Phe169, Trp171, Leu259, Arg329, Pro330, Gln396, Leu405, Trp407, Glu481, Ile561, His564, Met567
2	5.74	62.5	Phe169, Trp171, Glu174, Leu259, Arg329, Pro330, Gln396, Leu405, Trp407, Tyr473, Glu481, Ile561, His564, Met567
3	5.04	201.8	Asp328, Arg329, Pro330, Gln396, Trp407, Ala476, Ile479, Glu481, Arg528

## Appendix A Detailed results of docking simulations

Docking simulations were performed implementing *AutoDock Vina* (Trott & Olson, 2010 ▸) using default parameters. Therefore, 25 docking runs were performed for each ligand, while the binding energies and dissociation constants were estimated for each run. Afterwards, the 25 docking poses were clustered automatically by *YASARA* around hotspot conformations for cases in which the ligand r.m.s.d. is less than 5 Å. The run with the highest binding energy and the lowest dissociation constant (*K*_d_) in each complex was selected as representative of each cluster. Clustered results for each ligand are presented below. The clustered results of docking simulations were also evaluated based on previously determined structures of laccases in complex with various compounds (*i.e.* the structure of 2,6-DMP-bound *Ma*L; PDB entry 3fu7) and *in silico* studies of laccase substrate specificity (Mehra *et al.*, 2018 ▸). Docking poses in which the bound ligand was away from the substrate-binding site or the orientation of the ligand is not chemically favourable were rejected.

### A1. Docking results for l-ascorbic acid

The superposition of three clustered binding poses from *YASARA* is shown in Fig. 8 ▸. Of the 25 docked poses resulting from the simulation, 22 were clustered into the first group, three poses were clustered into the second group and only one was in the third group. The first clustered pose displays the most favourable binding energy and *K*_d_ value (Table 5 ▸) and is also presented in Section 3, while the second and third clustered poses of ascorbic acid are docked far from the substrate-binding site with large *K*_d_ values. The orientation and the binding position for these two cases indicate that substrate oxidation cannot occur and these results were rejected.

### A2. Docking results for 2,6-DMP

The 25 docking poses were all clustered into one group (Fig. 9 ▸; Table 6 ▸) that is presented in Section 3.

### A3. Docking results for ferulic acid

Solutions from 25 docking runs for the case of ferulic acid were clustered into three groups (Table 8 ▸) and the most favourable representatives in terms of binding energy are shown in Fig. 10 ▸. 13 poses were clustered into the first group, seven poses were clustered into the second group and four poses were clustered into the third group. The orientation of the docked molecules in the second and third clusters would not allow electron transfer from the O3 atom to His564 and therefore they were rejected.
