# Supplementary material for: Structure–function studies of a novel laccase-like multicopper oxidase from Thermothelomyces thermophila provide insights into its biological role
Source: Acta Crystallogr D Struct Biol. 2023 Jun 16;79(Pt 7):641–54. doi: 10.1107/S2059798323004175 (PMC10306062; doi:10.1107/S2059798323004175)
Supplement: Supplementary file 1 [file d-79-00641-sup1.pdf]

STRUCTURAL BIOLOGY

Volume 79 (2023)

Supporting information for article:

Structure–function studies of a novel laccase-like multicopper oxidase from *Thermothelomyces thermophila* provide insights into its biological role

Christos Kosinas, Anastasia Zerva, Evangelos Topakas and Maria Dimarogona

Table S1 Glycosylated residues of *Tt*LMCO1 and structural equivalent residues of other experimentally determined asco-laccase structures

***Tt*LMCO1** (7ZN6)	***MtL*** (6F5K)	***MaL*** (2Q9O)	***TaL*** (3PPS)	***BaL*** (3SQR)	***AnL*** (5LM8)
Asn37 *(1)	Asn61 (5)	Asn61(-)	Asn62 (-)	Asn55 (1)	Asn76 (-)
Asn65 (2)	Gly89	Gly89	Gly90	Gly83	Gly104

*Numbers in parentheses indicate the number of glycans modelled in each site while dashes indicate that the residue is not glycosylated. In all cases treatment with Endo-H has been done before crystallization.

Table S2 Closest structural homologues of *Tt*LMCO1 by DALI server (Holm, 2020)

***Tt*LMCO1** (7ZN6)	***Z*-score***	**RMSD (Å)**	**Nres**	***%ID***
C. pepo AsOx (1AOZ)	40.0	2.2	552	30
M. murashkinskyi Lac (5E9N)	39.5	2.1	495	28
T. hirsuta Lac (3FPX)	39.3	2.1	499	28
T. versicolor Lac (1KYA)	39.2	2.1	499	28
C. cinereus Lac (1A65)	37.0	2.2	504	27
Cerrena sp. Lac (5Z1X)	39.5	2.1	495	26
Z.mays Lac (6KLG)	38.2	2.6	547	26
**Lac15** (4F7K)	27.5	2.7	377	26
T.arenaria Lac (3PPS)	34.7	2.5	564	24
P. acidilactici MCO (6XIZ)	25.8	2.9	477	22

*Z-score is an optimized similarity score defined as the sum of equivalent residue-wise C_α - C_α distances among two proteins. A Z-score above 20 indicates that the compared structures are homologous.

Table S3 Interatomic distances and B-factors in the trinuclear copper site (TNC) and the T1 site.

***Tt*LMCO1**	**Distances (Å)**
T3a-T3b	4.83
T3a-T2	4.09
T3b-T2	3.98
T2-W1	2.77
T3a-O1	2.46
T3a-O2	2.54
T3b-O1	2.63
T3b-O2	2.35
	**B-factors ($Å^2$)**
T3a	28.47
T3b	31.60
T2	29.79
T1	28.06
W1	21.61
O1	32.03
O2	38.09

Values for the copper(II) are given in parentheses.

Table S4 YASARA docking calculations depicting the binding energy, dissociation constant and residues of *Tt*LMCO1 that contribute to ligand binding.

No	**Ligand**	**Binding Energy (kcal mol^{-1})**	**Dissociation constant (μM)**	**Contact Receptor Residues**
1	L-ascorbic acid	6.37	21.6	Phe169, Trp171, Glu174, Arg329, Gln396,Leu405, Trp407, Tyr473, Gly475,Ala476, Glu481,His564, Met567
2	2,6-DMP	5.46	99.5	Phe169,Trp171, Glu174, Leu259, Arg329, Pro330, Gln396, Leu405, Trp407, Tyr473, Ala476, Glu481, Ile561, His564, Met567
3	Ferulic acid	6.39	20.7	Phe169, Trp171, Leu259, Arg329, Pro330, Gln396, Leu405, Trp407, Glu481, Ile561, His564, Met567

Table S5 YASARA docking calculations depicting the binding energy, dissociation constant and residues of ascorbate oxidase of *Curcubita pepo* that contribute to ligand binding.

No	**Ligand**	**Binding Energy (kcal mol^{-1})**	**Dissociation constant (μM)**	**Contact Receptor Residues**
1	Ferulic acid	4.96	228.3	Trp163, Glu166, Arg285, Gln353, Trp362, Met436, Glu443,His445, His512, Met515

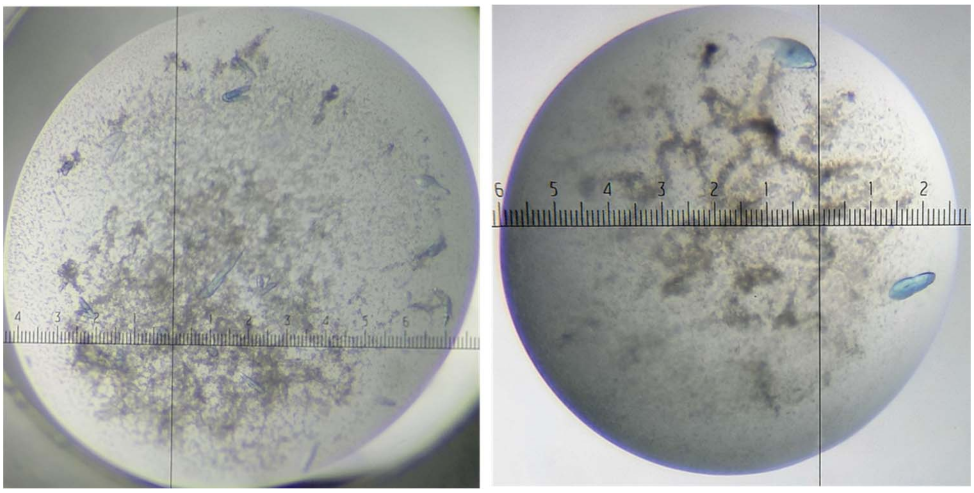

Figure S1 (a). *Tt*LMCO1 crystals grown in 0.2 M ammonium chloride, 0.1 M 2-(N-morpholino)ethanesulfonic acid (MES) pH 6.0, 20 % w/v PEG 6000 (b) Optimized *Tt*LMCO1 crystals in the same crystallization condition on pH 5.5.

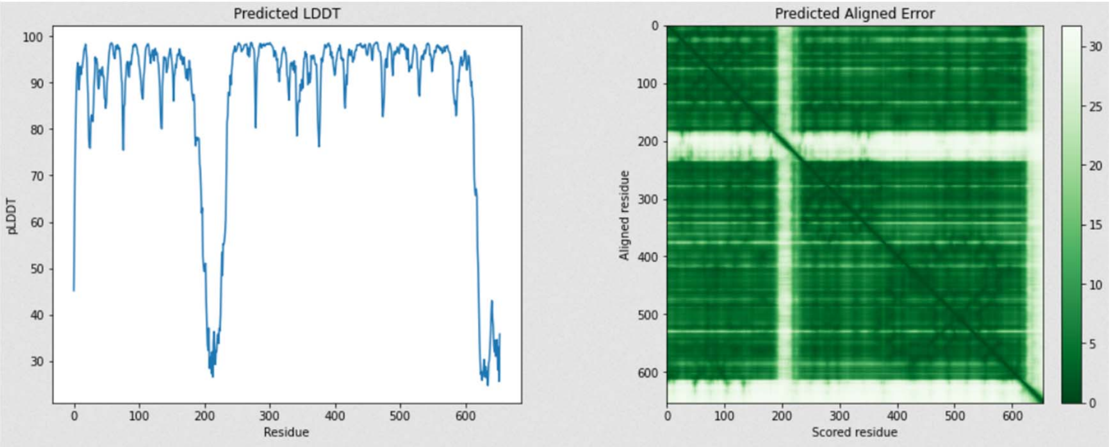

Figure S2 (*left*) AlphaFold model *Tt*LMCO1 calculations of per-residue predicted local distance difference test (LDDT) and (*right*) predicted align error as a distance error for every pair of residues.

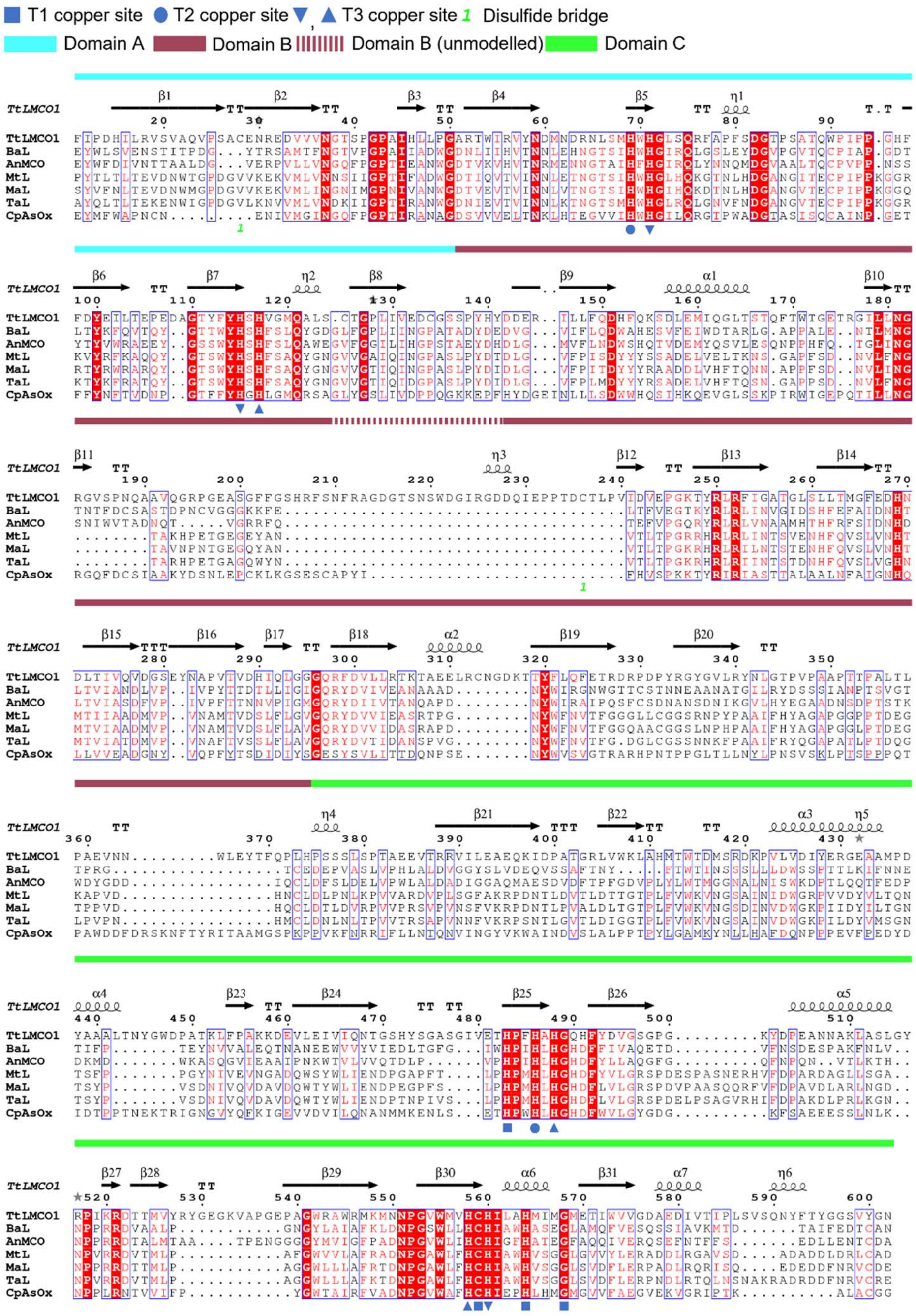

Figure S3 Multiple sequence alignment (including amino acids present in the crystal structure of *Tt*LMCO1) of *Tt*LMCO1, *Ba*L, *An*MCO, *Mt*L, *Ma*L, *Ta*L and *Cp*AsOx. Secondary structure elements of *Tt*LMCO1 are drawn as arrows (β-sheets) and helices. Residues that take part in T1, T2 and T3 copper site are highlighted. Identical and similar residues are printed in white on a red background and in red on a white background, respectively. The figure was prepared by ESPript 3.0 (Robert & Gouet, 2014).

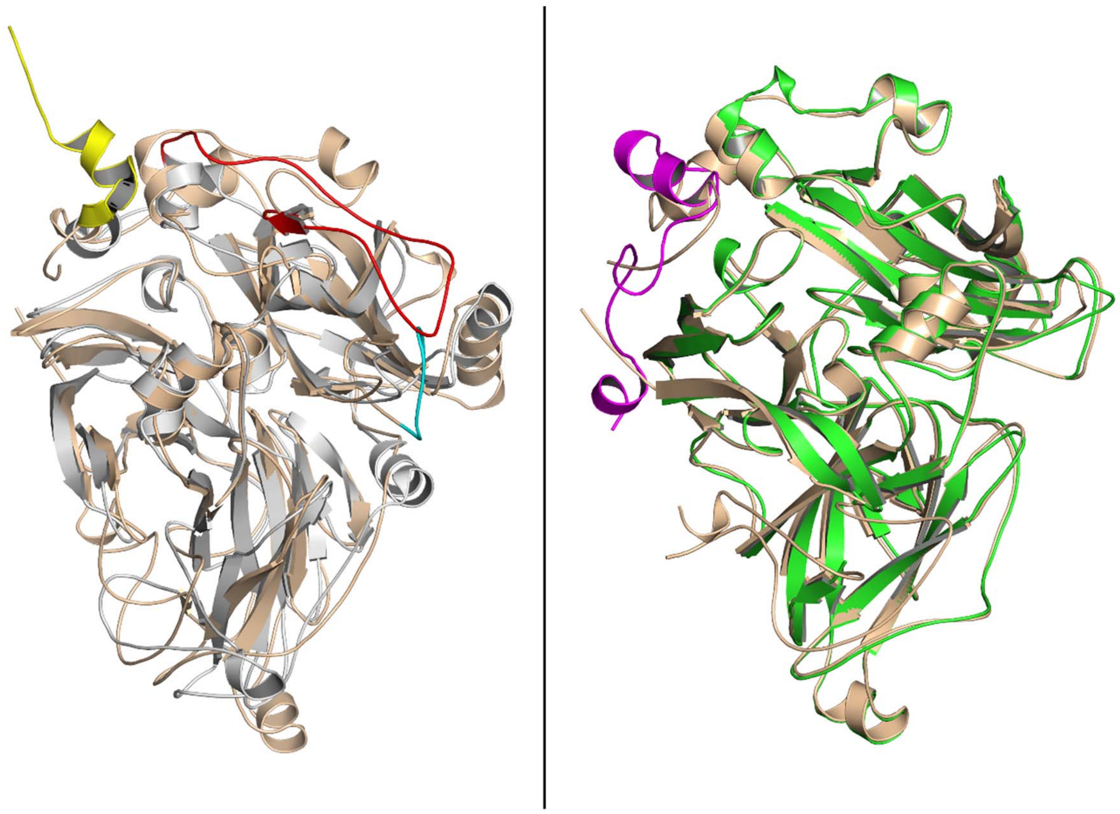

Figure S4 (*left*) Superposition of the final refined structure of *Tt*LMCO1 (wheat) with the ascorbate oxidase of *C. pepo* (silver) performed with PyMOL 2.0. RMSD over C_a atoms is estimated at 2.5 Å. Major differences at the C-terminal (yellow), the loop 382-426 (red) and the loop 529-539 (cyan) are highlighted in the *C. pepo* oxidase structure. (*right*) Superposition of the final refined structure of *Tt*LMCO1 with the prediction generated with AlphaFold 2.0 performed with PyMOL 2.0. RMSD over C_a atoms is estimated at 1.3 Å. There are no significant structural discrepancies between the two structures, with the exception of their C-terminal (depicted in magenta in AlphaFold 2.0 prediction).

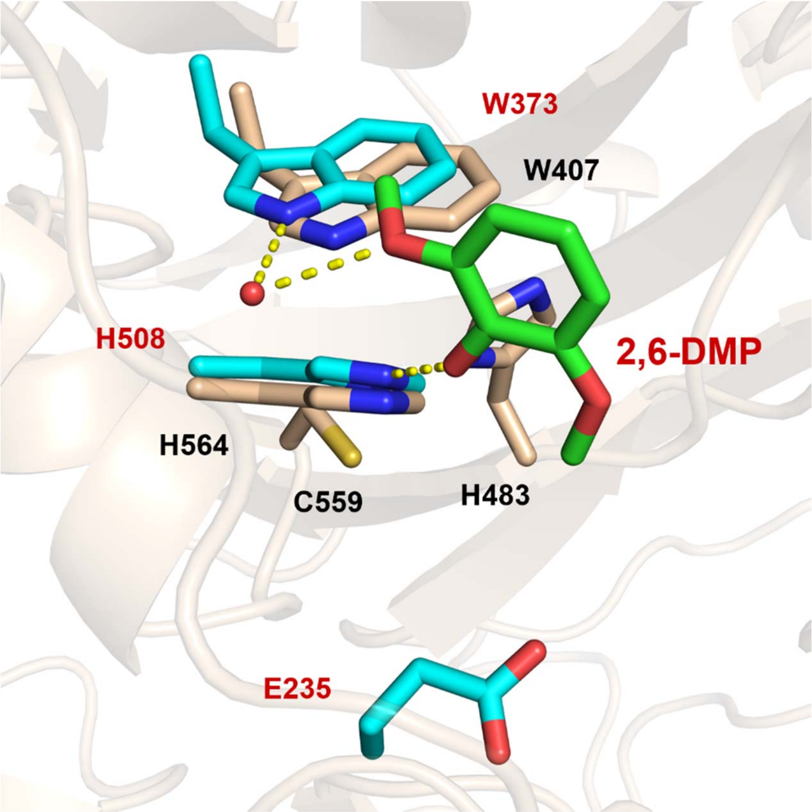

Figure S5 Superposition of the structure of 2,6 DMP-bound *Ma*L (3FU7) with *Tt*LMCO1. 2,6 DMP is shown in green sticks and side chains of residues Glu235, Trp273, His508 of *Ma*L structure are shown in cyan. A water molecule is shown as red sphere. In the MaL structure, 2,6-DMP interacts directly with His508 which coordinates the T1 copper. Also, Trp373 residue participates in substrate binding, assisted by a bridging water molecule, while Glu235 is also taking part in facilitating proton abstraction from phenolic compounds (Kallio *et al.*, 2009). His508 along with Glu235 form a polar site for phenolic substrate recognition, a characteristic that is typical for other asco-laccases too (Ernst *et al.*, 2018).

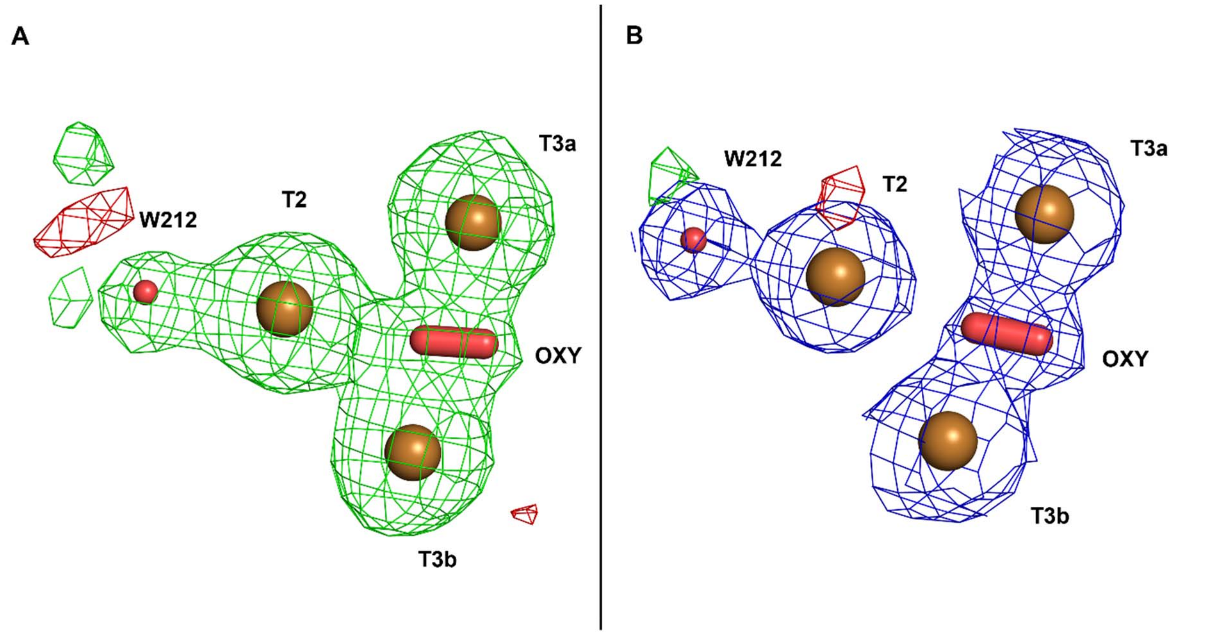

Figure S6 (**A**) Difference map (Fo-Fc), contoured at 3σ, prior to the inclusion of the copper atoms, the dioxygen ligand and the water molecule of the trinuclear copper site (TNC) in the model. Positive density is shown in green, negative in red. The subsequently modeled atoms of the TNC are also shown. (**B**) 2Fo-Fc (contoured at 1 σ) and Fo-Fc (contoured at 3σ) maps after the final refinement step. 2Fo-Fc map is shown in blue isomesh, while the Fo-Fc is shown as green for positive density and as red for negative. Negative density around the T2 copper is considered noise. All copper atoms are shown as brown spheres, dioxygen molecules as red sticks and the water molecules as a red spheres.

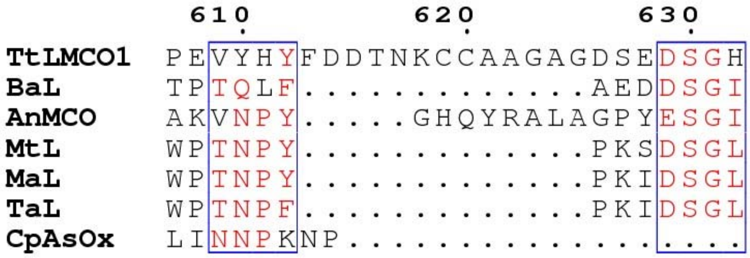

Figure S7 Multiple sequence alignment of the C-terminal amino acids of *Tt*LMCO1, *Ba*L, *An*MCO, *Mt*L, *Ma*L, *Ta*L and *Cp*AsOx. C-terminal sequence is conserved among the asco-laccases but not for the ascorbate oxidase from *C. pepo*.

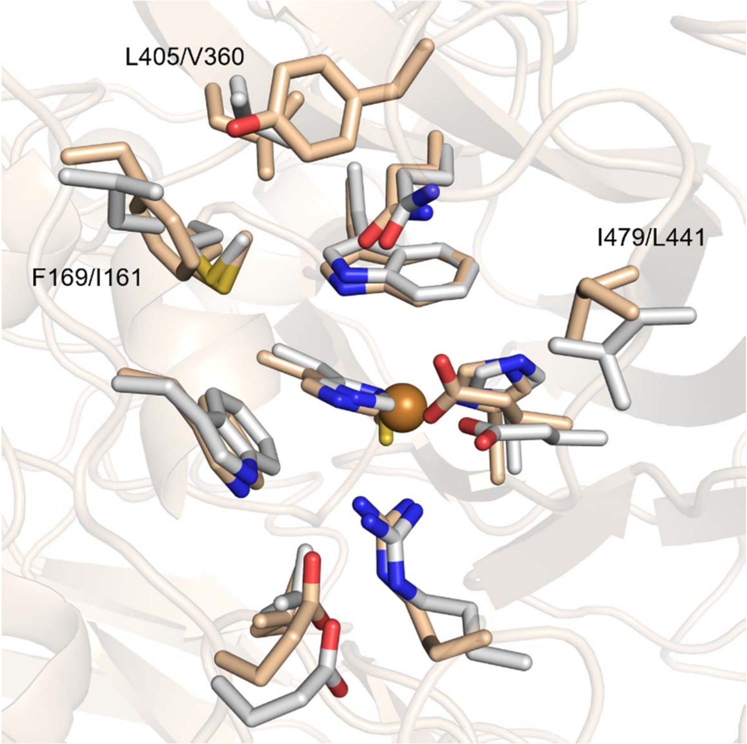

Figure S8 Superposition of residues forming the binding site of ascorbate oxidase of *Curcubita pepo* (silver sticks) with residues of *Tt*LMCO1 binding site (wheat sticks). Different residues are highlighted.
